# Nuclear Actin in Development and Transcriptional Reprogramming

**DOI:** 10.3389/fgene.2017.00027

**Published:** 2017-03-07

**Authors:** Shinji Misu, Marina Takebayashi, Kei Miyamoto

**Affiliations:** Laboratory of Molecular Developmental Biology, Faculty of Biology-Oriented Science and Technology, Kindai UniversityKinokawa-shi, Japan

**Keywords:** nuclear actin, actin polymerization, transcriptional reprogramming, chromatin remodeling, regulation of gene expression, actin-binding protein, stem cell, differentiation

## Abstract

Actin is a highly abundant protein in eukaryotic cells and dynamically changes its polymerized states with the help of actin-binding proteins. Its critical function as a constituent of cytoskeleton has been well-documented. Growing evidence demonstrates that actin is also present in nuclei, referred to as nuclear actin, and is involved in a number of nuclear processes, including transcriptional regulation and chromatin remodeling. The contribution of nuclear actin to transcriptional regulation can be explained by its direct interaction with transcription machineries and chromatin remodeling factors and by controlling the activities of transcription factors. In both cases, polymerized states of nuclear actin affect the transcriptional outcome. Nuclear actin also plays an important role in activating strongly silenced genes in somatic cells for transcriptional reprogramming. When these nuclear functions of actin are considered, it is plausible to speculate that nuclear actin is also implicated in embryonic development, in which numerous genes need to be activated in a well-coordinated manner. In this review, we especially focus on nuclear actin’s roles in transcriptional activation, reprogramming and development, including stem cell differentiation and we discuss how nuclear actin can be an important player in development and cell differentiation.

## Introduction

**FIGURE 1:**
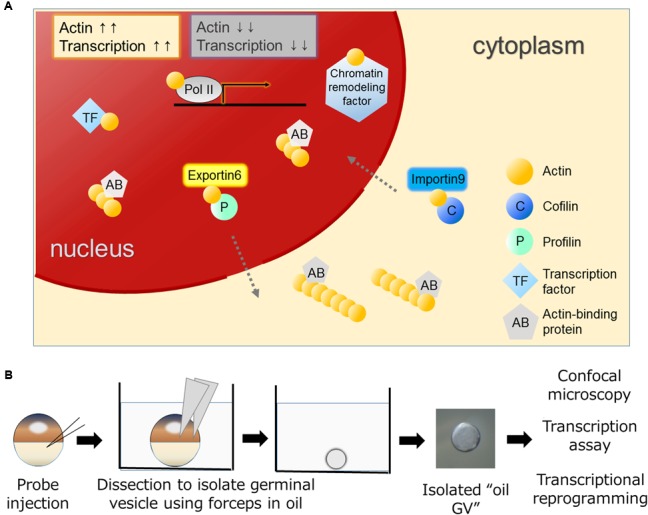
**Various functions of actin in the nucleus and the oil GV system to study nuclear actin. (A)** Nuclear actin binds to many nuclear proteins including transcription machineries, transcription factors, and chromatin remodeling factors. Nuclear actin-binding proteins such as actin nucleation promoting factors regulate nuclear actin polymerization. Nuclear concentrations of actin are regulated by IMPORTIN9 and EXPORTIN6 and high concentrations of nuclear actin are generally associated with high transcriptional activities. **(B)** Actin probes such as UtrCH-GFP mRNA are injected into *Xenopus* oocytes (Miyamoto et al., 2011). Germinal vesicles (GVs) filled with actin probes are isolated in mineral oil and these oil GVs can be observed by confocal microscopy to monitor nuclear actin dynamics. Oil GVs retain transcriptional activities, and when somatic nuclei are transplanted into the GVs, the injected nuclei undergo transcriptional reprogramming.

Eukaryotic cells have a globular, multifunctional protein called actin. Actin plays key roles in the cytoplasm by controlling a dynamic equilibrium between the monomeric and filamentous states for a variety of cellular processes, such as cell motility and adhesion. The amount of actin in nuclei is lower than that in the cytoplasm in most of cell types. Nevertheless, actin has also been found in the nucleus of various types of cells (Lestourgeon et al., 1975; Clark and Merriam, 1977; Clark and Rosenbaum, 1979; Pederson and Aebi, 2002). Nuclear actin has attracted a lot of attention, although its function has often been regarded as a controversial topic due to the technical difficulties of discriminating nuclear actin from cytoplasmic actin, which is highly abundant in cells. With the development of experimental tools, convincing evidence has been obtained that nuclear actin indeed does exist and plays crucial roles in many nuclear processes. For example, actin is found in all kinds of RNA polymerase complexes (Visa and Percipalle, 2010) and in a number of chromatin remodeling complexes (Zhao et al., 1998; Kapoor and Shen, 2014) (**Figure 1A**). It also binds to transcription regulators to control their activities (Grosse and Vartiainen, 2013).

**FIGURE 2:**
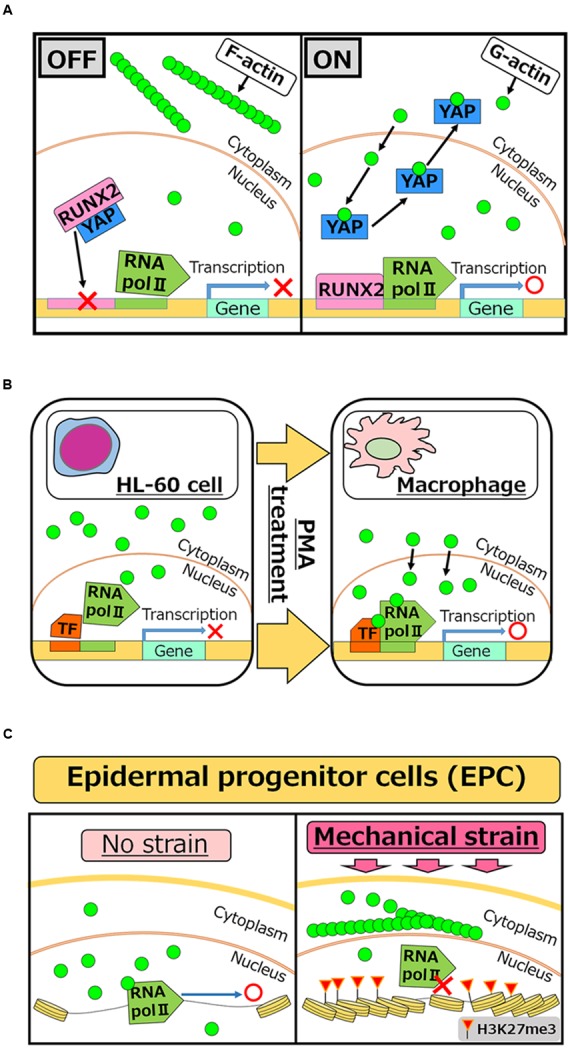
**Roles of nuclear actin in cell differentiation and lineage commitment. (A)** RUNX2 is a transcription factor which is required for activating the osteogenic gene, while interaction of RUNX2 with YAP protein inhibits its function as an activator (OFF). Increased concentrations of nuclear actin induce nuclear export of YAP, and therefore freed RUNX2 activates target genes for osteogenesis (ON) (Sen et al., 2015). **(B)** PMA treatment induces differentiation of HL-60 cells to macrophages. After the treatment, nuclear actin accumulates and induces transcription of genes related to macrophage activation (Xu et al., 2010). **(C)** When human EPCs are exposed to mechanical strain, actin polymerization is enhanced at the outer nuclear membrane, which in turn reduces the levels of nuclear actin. Reduced nuclear actin is associated with attenuated Pol II activities and the acquisition of H3K27me3, a repressive histone mark (Le et al., 2016).

Dynamic changes between globular (G-) and filamentous (F-) forms of nuclear actin affect these nuclear events. Nuclear actin polymerization is regulated by multiple mechanisms; more than 30 of actin-binding proteins that affect its polymerized states have been discovered in nuclei (Gieni and Hendzel, 2009; Castano et al., 2010; Kristo et al., 2016). It is of great interest to reveal how monomeric/polymeric actin is orchestrated by these actin-binding proteins during the above mentioned nuclear events. The regulation of the total amount of nuclear actin is another critical factor that affects activities of actin-mediated nuclear events. Nuclear actin is actively exported to the cytoplasm by EXPORTIN6 as the profilin-actin heterodimer (Stuven et al., 2003). Conversely, cytoplasmic actin together with cofilin is imported to the nucleus by IMPORTIN9 (Dopie et al., 2012). The active maintenance of nuclear actin levels is needed to support transcription (Dopie et al., 2012) (**Figure 1A**). In addition to these functions of actin to support fundamental nuclear processes, nuclear actin influences cellular phenotypes through transcriptional regulation and chromatin alternation, exemplified by transcriptional activation of the osteogenic genes in differentiating mesenchymal stem cells (MSCs; Sen et al., 2015) or by reorganization of heterochromatin during lineage commitment of epidermal stem cells (Le et al., 2016) (**Figure 2**). In this review, we summarize recent findings of functional roles of nuclear actin in transcriptional activation and chromatin remodeling. Currently, discovered functions of nuclear actin are executed in many different crucial cellular events such as transcription (de Lanerolle and Serebryannyy, 2011; Percipalle, 2013), transcriptional regulation (Grosse and Vartiainen, 2013), RNA processing (Percipalle et al., 2001; Visa and Percipalle, 2010), nuclear export (Percipalle et al., 2002), chromatin remodeling (Fenn et al., 2011; Kapoor et al., 2013; Kapoor and Shen, 2014), and nuclear reprogramming (Miyamoto and Gurdon, 2011; Miyamoto et al., 2011). We discuss how the nuclear functions of actin are related to development and cellular differentiation/dedifferentiation.

## Nuclear Actin and Transcriptional Activity

The implication of nuclear actin in transcription was first reported in the 1980s using amphibian oocytes (Scheer et al., 1984). Subsequently molecular evidence suggested that actin is contained in the transcription initiation complex of RNA polymerase II (Pol II) (Hofmann et al., 2004), bound to the C terminus domain of Pol II (Kukalev et al., 2005), and also bound to heterogeneous ribonucleoproteins (hnRNPs) and histone acetyltransferase PCAF during transcriptional elongation (Percipalle et al., 2003; Obrdlik et al., 2008). Furthermore, actin’s binding partners in the nucleus include not only the transcription machineries *per se*, but also the complexes of chromatin-remodeling factors. A variety of chromatin remodeling complexes contain actin, such as BAF (Zhao et al., 1998; Nishimoto et al., 2012), INO80 (Fenn et al., 2011; Kapoor et al., 2013), and SWR1 complexes (Kapoor and Shen, 2014). Additionally, it is also contained in histone-modifying complexes like NuA4 (Galarneau et al., 2000) and interacts with histone deacetylases (HDACs) (Serebryannyy et al., 2016). Examples of how nuclear actin plays a role in the chromatin remodeling factors have been shown in the BAF and INO80 complexes. The BAF complex, which belongs to the SWI/SNF family, slides and ejects nucleosomes in an ATP-dependent manner (Cairns, 2009). It can function as both transcriptional activators and repressors and its essential roles in development and reprogramming have been shown (Ho and Crabtree, 2010). Actin has been found as a monomer in the BAF complex and binding of β-actin to actin-related protein 4 (ARP4) modulates the activity of the complex (Nishimoto et al., 2012). The INO80 complex can evict and/or slide nucleosomes and is involved in telomere regulation, chromosome segregation, and DNA replication (Ho and Crabtree, 2010). In the INO80 complex, a monomer of nuclear actin is included in the INO80 subcomplex together with ARP4 and ARP8 (Fenn et al., 2011; Kapoor et al., 2013). Actin cannot bind to DNA directly, and instead actin-ARPs modules enable their binding to DNA (Gerhold et al., 2012). Requirement of monomeric actin for INO80-mediated chromatin remodeling has been demonstrated (Kapoor et al., 2013). Moreover, an increased nuclear actin concentration inhibits HDAC activities (Serebryannyy et al., 2016). Taken together, nuclear actin is involved in enhanced transcriptional states and active chromatin remodeling in general (**Figure 1A**). As a consequence, the amount of nuclear actin, regulated by IMPORTIN 9 (Dopie et al., 2012) and EXPORTIN 6 (Stuven et al., 2003), is crucial for transcription.

On top of these direct interactions between nuclear actin and chromatin proteins, actin regulates transcription levels by sequestering transcriptional activators (Kang et al., 2004) or repressors (Huang et al., 2011). A well-documented case is represented by serum response factor (SRF). SRF activity is regulated by binding of MAL, a co-activator of SRF transcription factor, to target genes (Vartiainen et al., 2007). However, if actin monomer binds to MAL, this MAL/actin binding induces not only inhibition of interaction between MAL and Pol II, but also nuclear export of the MAL/actin complex (Vartiainen et al., 2007). Increased actin polymerization results in reduced monomeric actin and consequently activates SRF target genes. Interestingly, nuclear actin polymerization is sufficient to activate SRF targets (Baarlink et al., 2013). In summary, multiple mechanisms related to nuclear actin lead to transcriptional activation and changes in polymerized states of nuclear actin often influence the transcriptional outcome.

## Nuclear Actin in Transcriptional Reprogramming

Differentiated cell states of somatic cells can be reversed to the undifferentiated one by nuclear reprogramming. Nuclear reprogramming of somatic cells entails global changes in transcriptional programs. This transcriptional reprogramming is achieved by nuclear transplantation of differentiated somatic cells to oocytes (Jullien et al., 2011). Especially when somatic nuclei are transplanted into the germinal vesicle (GV) – a giant nucleus – of the *Xenopus laevis* oocyte, the transplanted nuclei undergo widespread transcriptional reprogramming within 2 days without the need for cell divisions and new protein synthesis (Jullien et al., 2014). These characteristic features of the *Xenopus* oocyte nuclear transfer system enabled us to reveal several important factors and mechanisms underlying transcriptional reprogramming (Jullien et al., 2010, 2014; Miyamoto et al., 2011, 2013). Of note, nuclear actin and actin-binding protein play important roles during transcriptional reprogramming in oocytes (Miyamoto et al., 2011, 2013). *Xenopus* oocytes have advantages for studying nuclear actin for two main reasons. Firstly, GVs of *Xenopus* oocytes contain a large amount of nuclear actin due to the lack of EXPORTIN 6 (Bohnsack et al., 2006). This large amount of nuclear actin makes actin polymerization in nuclei easily visible. Secondly, GVs can be isolated in non-aqueous liquid, like mineral oil, without disrupting their transcriptional activities (**Figure 1B**) (Gall and Wu, 2010). Hereafter, we name these isolated GVs in mineral oil as “oil GV.” Oil GVs represent live purified nuclei and hence provide an optimal platform for nuclear actin research, since we do not have to consider the cytoplasmic function of actin (**Figure 1B**). Even transcriptional reprogramming can be induced in the oil GV system after nuclear transplantation of somatic nuclei into oil GVs (Miyamoto et al., 2011). Using the oil GV system, we have shown that perturbation of polymerized states of nuclear actin affects transcriptional reprogramming of *Oct4*, a key pluripotency gene; namely decreased actin polymerization results in failed activation of *Oct4* and increased actin polymerization is associated with enhanced *Oct4* expression (Miyamoto et al., 2011). Furthermore, we found WASF1 (also known as WAVE1), an actin-binding protein that can regulate actin polymerization, in GVs (Miyamoto et al., 2013). The knockdown of nuclear WAVE1 causes defects in transcriptional reprogramming (Miyamoto et al., 2013). These results indicate that both nuclear actin and nuclear actin-binding protein play roles in transcriptional reprogramming. Importantly, recent studies confirm nuclear actin’s contributions to transcriptional reprogramming using cultured cells. Forced expression of nuclear actin in Hela cells results in altered expression of approximately 2000 genes, including *Oct4* (Yamazaki et al., 2015). Moreover, nuclear F-actin, but not nuclear G-actin, plays a role in recruiting β-catenin to the *Oct4* locus for its activation (Yamazaki et al., 2016). Sadhukhan et al. (2014) revealed a nuclear role of Wiskott-Aldrich Syndrome Protein (WASP), an actin nucleation-promoting factor, in transcriptional activation during human T_H_ cell differentiation. They also found that WASP accelerates epigenetic modification, namely histone H3 lysine 4 trimethylation, independent of actin polymerization activities (Sadhukhan et al., 2014), in good agreement with WAVE1’s function in the frog system. Taken together, functional roles of actin and actin nucleation-promoting factor in the nucleus seem well-conserved across different species and different cell types although the amount of nuclear actin varies. It is also noteworthy that when cells are exposed to stimuli to change their stable states, nuclear actin tends to be formed. This is exemplified by transcriptional reprogramming (Miyamoto et al., 2011), DNA damage (Belin et al., 2015), mechanical strain (Le et al., 2016), and serum stimulation (Vartiainen et al., 2007; Baarlink et al., 2013). It would be therefore intriguing to observe dynamics of nuclear actin *in vivo* when cells are exposed to such stimuli during differentiation and development.

## Nuclear Actin in Development and Cell Differentiation

Considering nuclear roles of actin in transcriptional regulation, it is reasonable to speculate that nuclear actin is involved in cellular differentiation and development, which entail changes in transcriptional programs. Accumulating evidence indicates that this is indeed true at least in several different cell types. Induced accumulation of nuclear actin in MSCs causes transcriptional activation of the osteogenic genes osterix and osteocalcin, leading to the osteogenic phenotype. Mechanistically, YAP protein binds to and inhibits the osteogenic transcription factor RUNX2. The accumulation of nuclear actin induces nuclear export of YAP protein. As a result, RUNX2 is relieved from its repressive interaction with YAP and osteogenesis is enhanced in a RUNX2-dependent manner (Sen et al., 2015) (**Figure 2A**). Translocation of actin to nuclei is also observed when HL-60 cells differentiate toward macrophages after phorbol 12-myristate 13-acetate (PMA) treatment (Xu et al., 2010). In this case, nuclear β-actin is involved in transcriptional activation of target genes important for macrophage differentiation (**Figure 2B**). Another recent study shows that nuclear actin is involved in lineage commitment of epidermal progenitor cells (EPCs) through the regulation of the transcriptional activity (Le et al., 2016). Upon induction of mechanical strain on EPCs, enhanced actin polymerization at the outer nuclear membrane is observed, which in turn reduces the amount of nuclear actin, leading to suppression of RNA Pol II activity and the increase of histone H3 lysine 27 trimethylation (H3K27me3). This increased level of H3K27me3 is crucial for suppressing the expression of lineage-commitment genes (**Figure 2C**). In addition to these roles of nuclear actin in cell differentiation and lineage commitment, nuclear actin-binding proteins also serve as a key player for cell differentiation. In hematopoietic cells, WASP is localized in nuclei during T cell differentiation (Taylor et al., 2010). WASP supports histone H3K4 methyltransferase activity for transcriptional activation of T_H_1 genes, such as *IFNG* and *TBX21*, and is important for differentiation (Sadhukhan et al., 2014). It is noteworthy that nuclear roles of WASP seem uncoupled from its actin polymerization activity (Sadhukhan et al., 2014), although the actin-related protein 2/3 (ARP2/3) complex, which binds to WASP through the VCA domain, exists in nuclei (Yoo et al., 2007). Differential functions of other actin-binding proteins between the nucleus and the cytoplasm need to be determined in future studies.

Nuclear actin is found in female germ cells of *Xenopus laevis* (Clark and Merriam, 1977; Clark and Rosenbaum, 1979), *Drosophila* (Kelpsch et al., 2016), and avian species (Maslova and Krasikova, 2012). In the case of *Drosophila*, nurse cells and GVs contain nuclear actin during oogenesis and nuclear actin rod formation is regulated by Fascin (Kelpsch et al., 2016). In *Xenopus*, a large amount of nuclear actin in GVs contribute to mechanical stability of the giant nucleus (Bohnsack et al., 2006), stabilization of nuclear structure (Maslova and Krasikova, 2012; Feric and Brangwynne, 2013) and transcription (Scheer et al., 1984; Miyamoto et al., 2011). The amount of nuclear actin seems to decrease after oocyte maturation and fertilization since EXPORTIN 6 protein starts to be expressed after GV breakdown (Bohnsack et al., 2006). The abundance of nuclear actin and its functional roles in early embryonic development need further investigation. We have shown that an actin nucleation-promoting factor WAVE1, stored in the nuclei of *Xenopus* oocytes, is important for early embryonic development. Knockdown of maternal WAVE1 protein induces downregulation of *Hox* genes in gastrula embryos, which is rescued by nuclear WAVE1 expression (Miyamoto et al., 2013). In good agreement with our study, the requirement of nuclear N-WASP (neuronal Wiskott-Aldrich Syndrome Protein), another actin nucleation-promoting factor, and nuclear actin polymerization for *HoxB* expression upon retinoic acid treatment has been shown in cultured cells (Ferrai et al., 2009). However, it remains unknown to what extent WAVE1-regulated nuclear actin polymerization contributes to embryonic gene activation and early embryonic development since an isoform of WAVE1, which lacks the ARP2/3-binding VCA domain, can rescue *Hox* gene expression and embryonic development (Miyamoto et al., 2013). It is possible that nuclear roles of actin nucleation-promoting factor might be separated from their cytoplasmic functions, as is the case in WASP (Sadhukhan et al., 2014). Further research will reveal mechanisms of selecting actin-dependent/independent actions.

## Conclusion

Recent studies have uncovered the implication of nuclear actin in a variety of nuclear processes, including basal transcription (Percipalle, 2013), transcriptional activation (Grosse and Vartiainen, 2013; Miyamoto and Gurdon, 2013), chromosome movement (Chuang et al., 2006; Dundr et al., 2007; Mehta et al., 2010), structural integrity (Feric and Brangwynne, 2013), chromatin remodeling (Szerlong et al., 2008; Fenn et al., 2011; Nishimoto et al., 2012; Kapoor et al., 2013), apoptosis (Grzanka et al., 2014; Grzanka et al., 2015), cell cycle progression (Goyal et al., 2011; Spencer et al., 2011; Kalendova et al., 2014), DNA repair (Andrin et al., 2012; Belin et al., 2015) and nuclear assembly (Krauss et al., 2003). These various features of nuclear actin could be related to cellular phenotypes during or after differentiation. It is likely that roles of nuclear actin will expand even more in the near future. For detailed understanding of nuclear actin, especially in the context of actin polymerization, it is critically important to develop new probes and systems to monitor nuclear actin dynamics (Belin et al., 2013; Plessner et al., 2015) and to distinguish nuclear and cytoplasmic actin. It is equally important to use genome-wide or a large-scale approaches to identify interactome of nuclear actin (Rohn et al., 2011; Samwer et al., 2013; Dopie et al., 2015). Structural insights into nuclear actin (Cao et al., 2016) will also accelerate our understanding. Finally, biological relevance of nuclear actin formation *in vivo* cells and/or in the context of pathogenesis warrants further investigation.

## Author Contributions

All authors listed, have made substantial, direct and intellectual contribution to the work, and approved it for publication.

## Conflict of Interest Statement

The authors declare that the research was conducted in the absence of any commercial or financial relationships that could be construed as a potential conflict of interest.
